# eIF4A inhibition: ready for primetime?

**DOI:** 10.18632/oncotarget.26268

**Published:** 2018-10-30

**Authors:** Tyler A. Cunningham, Eli Chapman, Jonathan H. Schatz

**Affiliations:** Jonathan H. Schatz: Sylvester Comprehensive Cancer Center, University of Miami Miller School of Medicine, Miami, Florida, USA; Division of Hematology, Department of Medicine, University of Miami Miller School of Medicine, Miami, Florida, USA

**Keywords:** cap-dependent translation, experimental therapeutics, eIF4A, drug discovery, signaling

**Figure 1:**
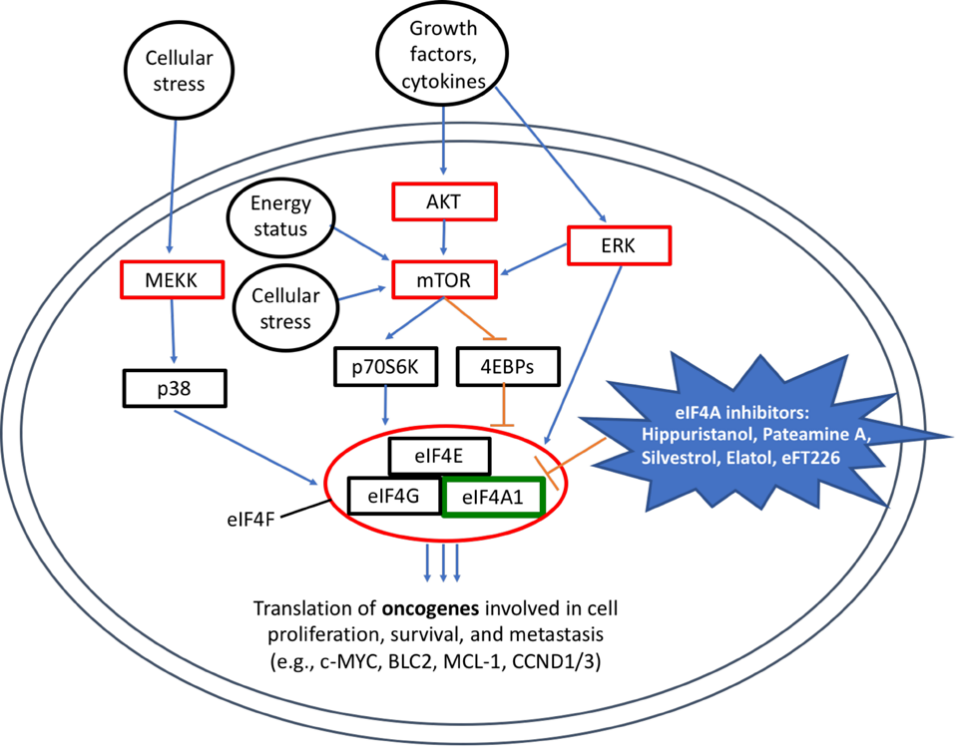
Many pro-oncogenic signaling pathways converge on cap-dependent translation and can be inhibited by targeting eIF4A

Targeted signaling inhibitors have improved treatment options for many cancers, but resistance mediated by redundancies in pathways limits clinical efficacy [1]. Activation of eIF4F, the complex that carries out cap-dependent translation initiation, is a convergence of multiple upstream oncogenic signals and drives resistance to targeted inhibitors (Figure 1) [2]. The complex comprises the cap-binding protein eIF4E, the scaffold protein eIF4G, and eIF4A, a DEAD-box RNA helicase responsible for unwinding secondary structures in mRNA transcripts. In 2004, the Pelletier group employed a bicistronic mRNA reporter to screen for eukaryotic protein translation inhibitors [3]. The reporter permitted multiplexed screening that distinguished between activity against cap-dependent initiation, IRES-mediated initiation, and elongation/termination. A series of compounds with specific activities against cap-dependent initiation emerged and were characterized in detail in the decade that followed. These include most notably the natural compounds hippuristanol, pateamine A, and the rocaglate silvestrol [4, 5]. All these compounds have the same molecular target, eIF4A, the ATP-dependent enzymatic core of the eIF4F complex. This activity is associated with potent anti-tumor activity, best characterized for silvestrol. Silvestrol binds to free (non-complexed) eIF4A and increases its affinity for mRNA in a sequence-specific manner, leading to a stable ternary complex, and preventing enzymatic unwinding of the secondary structure. G-quadruplexes and polypurine stretches of RNA in the 5’UTR exhibit greater binding by eIF4A-silvestrol and other rocaglates, and there is decreased translation of mRNAs harboring these characteristics [6, 7]. *In vivo* studies, including our group’s recent work (detailed below), have established a therapeutic window for targeting the eIF4F complex [8, 9]. Overall, this body of work has clearly established eIF4A as a “privileged” target in cancer therapeutics preclinically, but to date no compound with this activity has made it to clinical evaluation. That may at last be about to change.

Biopharmaceutical company eFFECTOR Therapeutics is at the frontier of drug development for targeting translation with plans to begin phase I clinical trials (early 2019) using compound eFT226, a selective translation regulator. Meeting abstracts indicate that *in silico* models including ab initio ligand-based methods and analysis of crystal structures were used in the compound design. Preclinical data on eFT226 is not yet published, but a poster presented at the American Society of Hematology meeting in 2017 suggests a mechanism of action similar to the rocaglates, in which the compound increases the affinity of eIF4A for the mRNA in a sequence specific manner. While these results suggest a promising candidate, we must await peer-reviewed published data for thorough evaluation of the compound’s mode-of-action. If able to successfully enter phase 1, however, eFT226 could be the first selective regulator of translation initiation to begin human trials and could set the stage clinically for a new class of anticancer therapeutics [10].

We took a different approach to eIF4A, exploiting its established status as a privileged target and carried out pilot target-based screening. Our recently published identification and characterization of the marine-derived natural compound elatol as a potent inhibitor of eIF4A with *in vivo* antitumor activity establishes proof-of-principle of our target-based approach [9]. We used an initial cell-free screen to identify inhibitors of eIF4A ATPase activity. Based on its relatively potent antitumor activities, elatol was further characterized and found to inhibit eIF4A helicase activity, and showed no activity against other tested ATP-hydrolyzing enzymes, including other DEAD box helicases, or kinases. Although less potent than silvestrol, elatol exhibited antitumor activity across a panel of cancer cell lines, with leukemias and lymphomas most sensitive overall. Assessment of effect on translation inhibition revealed that elatol preferentially inhibits cap-dependent translation compared to IRES-mediated translation, and experiments using elatol to treat tumor-bearing mouse xenografts established a therapeutic window *in vivo*. Off-target effects, including induction of an integrated stress response for unclear reasons, and limited opportunities for optimization through medicinal chemistry have halted further work on elatol specifically. However, this work provides proof of principle for a pipeline to identify eIF4A inhibitors starting with a cell-free target-based approach that measures inhibition of ATPase activity and to further characterize these hits *in vitro* and *in vivo*.

Moving forward, we are carrying out high-throughput screening employing an optimized platform that simultaneously assesses all three paralogs of eIF4A (eIF4A1-3). eIF4A1 is the key player in cap-dependent initiation, and our work to date suggests identifying off-target activity against the closely homologous but biologically distinct paralogs eIF4A2 and eIF4A3 will be important in optimizing drug candidates. Compounds considered hits in our cell-free ATPase inhibition assay are taken through a series of biochemical, cell-based, and when appropriate *in vivo* verification steps. These include eIF4A1 helicase inhibition, binding analyses with mutagenesis confirmation, assessment of preference for inhibition of cap-dependent vs. IRES-mediated translation, and of course assessments of anti-tumor potency. *In vivo* experiments are used to determine maximum tolerated dose of compounds and their effect on tumor growth in xenograft and immunocompetent tumor models. Our high throughput, target-based screen and downstream confirmatory drug pipeline with mechanistic studies will enable us to screen thousands of compounds and to identify eIF4A1-specific protein translation inhibitors. With cancer resistance and relapse to targeted signaling inhibitors still major clinical issues, the emergence of an eIF4A1-targeted clinical candidate in our opinion reveals a substantial new opportunity for cancer drug development. We and others are committed to seeing this treatment approach finally achieve testing in cancer patients. Our focus is on establishment and ongoing refinements of a pipeline able to narrow in on the most promising novel inhibitors of eIF4A-mediated protein translation. Targeting regulatory mechanisms of protein translation, the convergence point of many oncogenic signaling pathways, is a rational approach to killing cancer cells with functionality of many oncoproteins and pro-survival factors being lost or diminished.
